# ﻿Freshwater Mollusca of Kazakhstan (Central Asia): species composition and estimation of taxonomic richness

**DOI:** 10.3897/zookeys.1250.156288

**Published:** 2025-08-26

**Authors:** Ivan O. Nekhaev, Anel A. Ishayeva, Maxim V. Vinarski, Evgeniy S. Babushkin

**Affiliations:** 1 Institute of Zoology, Almaty, Kazakhstan, 050060, Al-Farabi Ave., 93, Almaty, Kazakhstan Institute of Zoology Almaty Kazakhstan; 2 Saint Petersburg State University, Saint Petersburg, 199034, Universitetskaya Emb., 7-9, Saint Petersburg, Russia Saint Petersburg State University Saint Petersburg Russia; 3 Surgut State University, 1 Lenina Ave., 628403, Surgut, Russia Surgut State University Surgut Russia; 4 Tyumen Scientific Center, Siberian Branch, Russian Academy of Sciences, 86 Malygina Str., 625026, Tyumen, Russia Russian Academy of Sciences Tyumen Russia

**Keywords:** Bivalvia, Central Asia, endemism, freshwater biodiversity, Gastropoda, taxonomy

## Abstract

Central Asia, including its freshwater basins, belongs to the Palearctic realm but exhibits significant faunal heterogeneity with localised hotspots of endemism. While several Central Asian countries have been the subject of malacological reviews, Kazakhstan, the largest country of the region, lacks a comprehensive and taxonomically updated species list. The aim of this study is to consolidate all available data on Kazakhstan’s freshwater molluscs, critically analyse their species composition, and assess the level of endemism in this group across the country. Based on the analysis of museum collections and literature sources, we estimate the species richness of freshwater molluscs in Kazakhstan at 87 species (55 Gastropoda and 32 Bivalvia), with an expected total of 93 species. Overall, species richness declines from the forest-steppe zone in the north and northeast toward the arid and mountainous regions in the south and southwest. Six potentially endemic species were identified; however, the taxonomic status of some requires further verification. The freshwater malacofauna of Kazakhstan has been affected by recent invasions, with at least seven species classified as neo-biotic, and for some, we hypothesise a recolonisation of habitats lost during the Pleistocene.

## ﻿Introduction

The territory of Central Asia, including its freshwater basins, belongs, from a biogeographer’s point of view, to the Palearctic realm. However, within this region a significant heterogeneity in the distribution of freshwater fauna is found, with local ‘hotspots’ of endemism that have been identified by various authors (Starobogatov 1970; Izzatulaev and Starobogatov 1985; Abell et al. 2008; Leroy et al. 2019). The accurate assessment of taxonomic diversity and the identification of distribution patterns of freshwater animals of this region are hindered by the insufficient knowledge of the fauna and high level of taxonomic impediment in many areas of Central Asia. Despite a long history of research, the freshwater fauna of this territory, including molluscs (snails and bivalves) remains relatively poorly studied. There are review papers on malacofauna of only selected countries of the Central Asian region in the literature: Afghanistan (Likharev and Starobogatov 1967), Turkmenistan (Starobogatov 1994), Kyrgyzstan (Glöer et al. 2014) and Mongolia (Vinarski et al. 2017a). Many species records published in the older papers are based on an outdated taxonomy and / or nomenclature and must be carefully revised.

The largest country of Central Asia, the Republic of Kazakhstan, still lacks even a non-annotated list of freshwater Mollusca inhabiting its territory. There are a few regional species lists, often based on isolated studies (Butenko 1967; Belyakova 1980; Frolova 1984; Vinarski et al. 2006). All of them are now taxonomically outdated. No effort has yet been made to consolidate this data into a comprehensive overview, with taking into account all taxonomic novelties proposed in recent times. The aim of our study is to accumulate all existing information on freshwater molluscs of the territory of Kazakhstan and to critically analyse it. We aimed to estimate the species richness of this group in the country and predict their actual diversity based on a statistical approach. Besides, the compiled list of species has allowed us to identify some patterns of distribution of freshwater molluscs across the country and to identify the level of endemism.

### ﻿A brief history of molluscan research in Kazakhstan

The fauna of freshwater molluscs in Kazakhstan has been studied for a long time, but most often these studies were sporadic or focused on individual regions of the country. Peter-Simon Pallas (1741–1811) was the first naturalist who reported data on molluscs living in the territory which nowadays belong to the Republic of Kazakhstan. During his famous travels throughout the southern provinces of Russian Empire (1769–1774), Pallas visited West Kazakhstan in 1769 and reported scarce data on molluscs of the Ural River basin and the northeastern part of the Caspian Sea (Pallas 1771). The most important among his findings was the new species, known today as zebra mussel, *Dreissena polymorpha* (Pallas, 1771), discovered by Pallas in the Ural River basin and in the Caspian Sea (see Vinarski 2023 for details). In the 19^th^ century, most information on the freshwater Mollusca of Kazakhstan was collected by separate expeditions that visited this area from time to time. The first attempts to synthesise this data were made by Western European malacologists, who worked with samples brought back to Europe by Russian explorers (von Martens 1874, 1882; Westerlund 1897). There was no modern administrative division at that time, and the distribution of continental Mollusca was often described in broad terms such as “Central Asia” or “Turkestan.” Some of the localities were obviously situated on the territory of present-day Kazakhstan. The only local collector of molluscs known in the 19^th^ century was a German physician Friedrich August Gebler (1781–1850), who lived in Barnaul City (South Siberia). He travelled extensively and studied malacofauna of East Kazakhstan, including the Rudnyi Altai Mts. and Lake Zaisan (Vinarski 2009). Gebler donated some of his shell samples to Alexander von Middendorff, one of the most prominent explorers of Mollusca in Russia of the 19^th^ century. Middendorff used these specimens to compile his monograph on molluscs of Northern Asia (von Middendorff 1851).

One of the most interesting episodes of the malacological exploration of Kazakhstan was the expedition conducted by American malacologist Alan Mozley (1904–1971). He visited the USSR in 1931–1932 and made an extensive travel across Southern Siberia, visiting, among other, some regions of Northern and Central Kazakhstan (Mozley 1936; Vinarski 2020). Mozley described a few species of freshwater molluscs with the type localities situated in Kazakhstan: Lymnaea (Galba) palustris saridalensis Mozley, 1934, Lymnaea (Galba) palustris kazakensis Mozley, 1934, Lymnaea (Galba) palustris draverti Mozley, 1934, and Planorbis (Spiralina) johanseni Mozley, 1934. Vinarski (2020) illustrated and discussed the type specimens of these four species.

Zhadin (1933, 1952) was the first author who made a synthesis of all faunal and distributional information on the freshwater molluscs of the whole ex-USSR territory, including Kazakhstan. The latest of his monograph (Zhadin 1952) provides the first estimate of species richness of this group in drainage basins represented in Kazakhstan. In Zhadin’s survey, freshwater regions of Kazakhstan were mainly represented by the basins of the Ural River (44 species), the Syr Darya River (20 species), and Lake Balkhash (13 species). However, it is not entirely clear how many species were recorded directly from the territory of modern Kazakhstan, since Zhadin did not precisely define and locate his sampling sites, and part of the drainage basins studied by him is located outside the borders of the Republic. For example, the list of species for Syr Darya provided by Zhadin (1952) also included species known only from Lake Issyk-Kul, situated in the neighbouring Republic of Kyrgyzstan.

Later, more detailed but locally focused faunal works appeared, covering individual regions, in particular, the upper Irtysh basin (Krivosheina and Starobogatov 1973; Krivosheina 1979) and Northern Kazakhstan (Frolova 1984). Lazareva (1967, 1968) focused on the Lymnaeidae fauna of Kazakhstan during her PhD project. Materials on the distribution of representatives of individual families and genera of freshwater molluscs in Kazakhstan have been presented in numerous taxonomic summaries covering more extensive territories of the Palearctic (Starobogatov 1970; Kruglov 2005). It is important that a significant part of faunal studies made by Kazakhstan researchers during the Soviet time was aimed at identifying and describing the parasitic fauna of molluscs, while information about the species composition of the molluscs themselves was often of a secondary importance (Butenko 1967; Smirnova 1967; Belyakova 1975, 1980). A lot of faunal information have been scattered among papers published by hydrobiologists, who often provided non-annotated species lists of all benthic macroinvertebrates, including Mollusca (Tseeb 1940; Stuge et al. 2009; Palatov and Vinarski 2022).

Since 2000, the northern and central parts of Kazakhstan were studied by a group of malacologists who then worked in Omsk, Russia. The aim of their research was the freshwater malacofauna of Western Siberia as a whole, including the parts of this region that belong to Kazakhstan. As a result, a series of publications, devoted to the entire Western Siberian malacofauna (Vinarski et al. 2007; Andreeva et al. 2010) or especially to particular groups of freshwater Mollusca of Kazakhstan (Lazutkina et al. 2012; Andreev et al. 2018), was published. However, even these relatively recent works are somewhat outdated due to intensive revisions of several families of freshwater snails and bivalves that have appeared during the last years (e.g., Aksenova et al. 2018, 2024; Bespalaya et al. 2024). In this paper, we take into account all novelties in freshwater Mollusca taxonomy published since 2018.

## ﻿Materials and methods

### ﻿Study area

**Figure 1.**
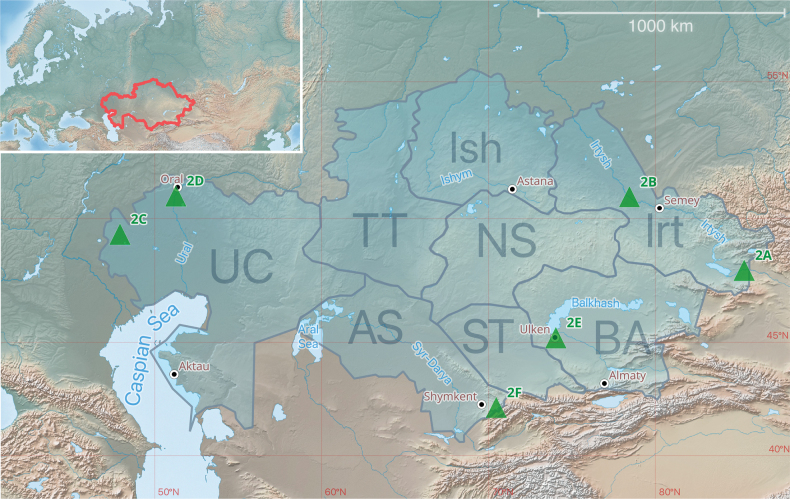
An overview map of Kazakhstan and the distribution of its primary drainage basins. Key: AS – Aral-Syr Darya, BA – Balkhash-Alakol, Irt – Irtysh, Ish – Ishim, NS – Nura-Sarysu, ST – Shu-Talas, TT – Tobol-Turgay, UC – Ural-Caspian. Locations depicted in Fig. 2 are marked with green triangles. For descriptions of location codes (2A–2F) see Fig. 2.

**Figure 2.**
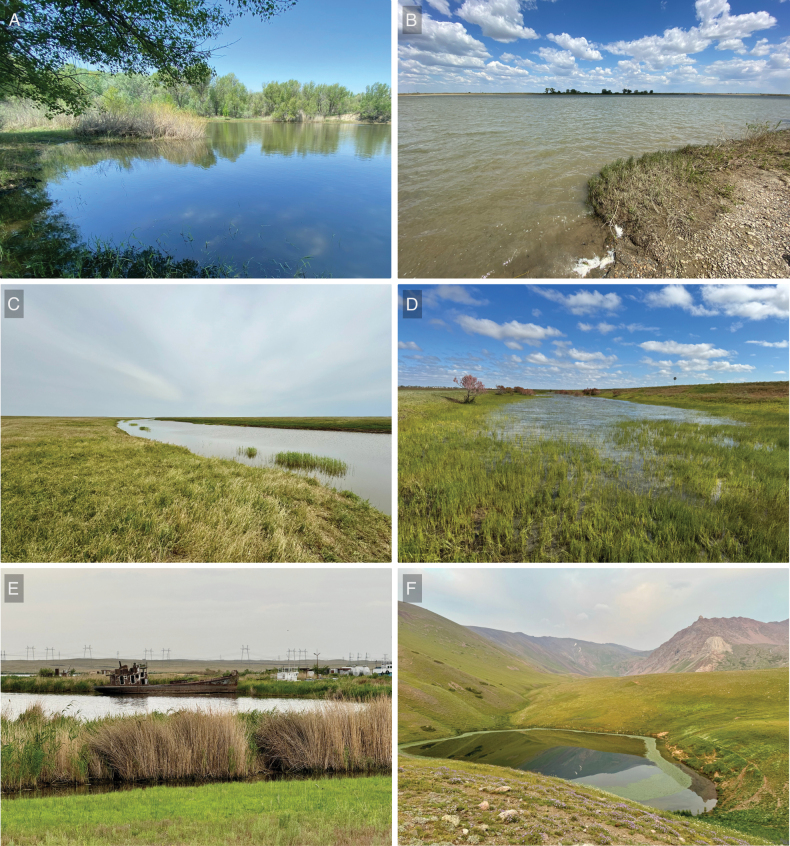
Selected habitats of freshwater molluscs in Kazakhstan. A. Upper Irtysh River, forest-steppe; B. Irtysh River near Pavlodar City, forest-steppe; C. A river in the northwestern part of Kazakhstan, steppe zone; D. A floodplain waterbody near Oral City, forest-steppe; E. The freshwater part of the Balkhash Lake near Ulken settlement, steppe; F. Mountain landscape in Talas Alatau, 2800 m a.s.l. The locations of the habitats are shown in Fig. 1.

Kazakhstan is located at the centre of the Eurasian continent and is the ninth-largest country in the world, with an area of approximately 2.7 million km^2^ (Fig. 1). Most of its territory consists of lowlands and plains. In the east and southeast, Kazakhstan is bordered by the mountain ranges of the Tien Shan and Altai. The country encompasses five major natural zones: forest-steppe, steppe, semi-desert, desert, and mountains (Pilifosova et al. 1997) (Fig. 2).

The climate of Kazakhstan is distinctly continental. In the northern regions, within the forest-steppe zone, the average number of days per year with subzero temperatures can reach up to 186, whereas in the southern regions (excluding mountainous areas) dominated by desert landscapes, this value is less than 80 (Salnikov et al. 2023). Maximum daily temperatures exceeding 25 °C are observed on average for approximately 50 days in the north and 140 days in the south. The annual average precipitation ranges from 350 mm in the north to 158 mm in the arid southern deserts, with a maximum of over 600 mm recorded in the Tien Shan Mountain regions (Salnikov et al. 2023). Currently, Kazakhstan is experiencing a trend of rising average temperatures and increasing aridification of many natural landscapes (Pilifosova et al. 1997; Salnikov et al. 2023).

The hydrological network of Kazakhstan is relatively underdeveloped. The country’s largest rivers – the Irtysh, Ural, and Syr Darya – are located primarily along its borders, while the central regions are characterised by the dominance of smaller watercourses and the inland flow basins. The largest lakes in Kazakhstan are brackish or partially saline, such as the Caspian Sea and Lake Balkhash. The territory of the country is divided into eight major drainage basins: Ural-Caspian, Tobol-Turgay, Ishim, Aral-Syr Darya, Shu-Talas, Nura-Sarysu, Irtysh, and Balkhash-Alakol (Tyumenev 2008) (Fig. 1).

### ﻿Data sources and analyses

We used two primary sources of information in order to compile the species list of freshwater Mollusca and identify their distribution patterns: museum collections and published records.

The following museum collections were examined:

Laboratory of Macroecology and Biogeography of Invertebrates, Saint Petersburg State University, Russia **(LMBI).** This collection contains many lots of Kazakhstan freshwater Mollusca which were sampled by Nikolay Andreev, Svetlana Andreeva, Maxim Vinarski, and his colleagues since the late 1970s. These collections were a critical data source, comprising 938 lots examined in our study.

Zoological Institute of the Russian Academy of Sciences, Saint Petersburg, Russia**(ZIN).** During its two centuries long history, ZIN has traditionally served as a repository for type materials of mollusc species, whose type localities are situated within the former Russian Empire and later the former USSR territory. ZIN collection includes numerous samples of freshwater gastropods and bivalves collected in territory of the modern Kazakhstan since the mid-19^th^ century. The oldest ones originated from Gebler’s collection – these snails were donated by him to Alexander von Middendorff in the 1840s. Examination of the ZIN collection in this institution primarily facilitated the reconciliation of earlier identifications with the modern taxonomic framework. We examined the representatives of most nominal species of the Kazakhstan freshwater molluscs available in ZIN, including their type material. However, the museum lots that represent common species (e.g., *Lymnaea stagnalis* s.l., *Radix auricularia*) were usually not re-examined unless it was necessary to confirm their occurrence in Kazakhstan or a specific drainage basin. In total, 431 lots were re-examined.

Institute of Zoology of the Republic of Kazakhstan, Almaty, Kazakhstan**(IZK).** Despite the long history of Mollusca collection of this institution, a significant portion of the material deposited by previous researchers (Z. Izzatullaev, Yu.V. Belyakova-Butenko, K.K. Uvalieva, T.S. Rymzhanov, and others) was lost during the 1990s–2000s. Currently, the IZK freshwater mollusc collection chiefly consists of specimens collected by the authors of this study, Ivan Nekhaev and Anel Ishayeva, since 2022. In total, 203 museum lots from this collection were examined.

Additionally, the data on some mollusc specimens kept in the Gothenburg Natural History Museum, Gothenburg, Sweden (**GNHM**) were included in the present research. Some samples of the family Sphaeriidae collected in Kazakhstan and inspected during this research are held in the personal collection of Evgeniy Babushkin and will be transferred to the ZIN collections in the future. A complete list of collection lots used in this study and their locations is provided in Suppl. material 1.

In addition to museum collections, 967 freshwater mollusc records were obtained from the literature. A total of 47 publications and dissertation abstracts were analysed, from which data on mollusc occurrences were extracted. Most of the reviewed works focus on the cataloguing of freshwater malacofauna of particular regions or on the trematode fauna of freshwater Mollusca. Additionally, we examined a series of papers published by hydrobiologists. They often contain useful records of mollusc species in various waterbodies but are rarely accompanied by descriptions (or illustrations) of shells, which creates a problem of verification of the provided information. All used molluscan records from published sources and a full list of these sources are provided in Suppl. material 1.

Molluscan records lacking precise coordinates, whether from museum collections or the literature, were georeferenced to the highest possible accuracy based on the descriptions of sampling regions. Using these coordinates, mollusc occurrences were associated with the primary drainage basins (Fig. 1). In regions with numerous brackish water habitats, the limited primary data often prevent determining whether a certain habitat was a part of brackish or freshwater environment. In such cases, we included in the species list only those molluscs belonging to typically freshwater taxa, such as basommatophoran snails and representatives of the families Valvatidae, Bithyniidae, Sphaeriidae, and Unionidae. Conversely, taxa primarily associated with brackish water (e.g., most species of the families Cardiidae and Hydrobiidae sensu lato) were excluded unless their presence in freshwater habitats was explicitly confirmed.

### ﻿Taxonomy

In the 1980s–2000s, most of research of freshwater Mollusca in the former USSR was conducted according to the taxonomic approach developed by Yaroslav Starobogatov and his pupils and followers. This approach was based on recognising the validity of numerous species that differed only slightly in their shell proportions and shape (the so-called “comparative method”; see Shikov and Zatravkin (1991) and Vinarski and Kantor (2016) for details). Numerous subsequent studies employing anatomical features, molecular markers, or analyses of shell variation in large mollusc samples have demonstrated the lack of real differences among a majority of the “comparatory” species (Graf, 2007; Bolotov et al. 2013; Vinarski et al. 2017b, 2021b; Bogatov et al. 2018; Bespalaya et al. 2024). Moreover, rarely species of freshwater Mollusca delineated by means of the comparative method have molecular support and represent real evolutionary units (Vinarski et al. 2015a; Aksenova et al. 2018, 2024; Bespalaya et al. 2024).

Many nominal taxa of aquatic snails and bivalves delineated under this approach can be relatively easily associated with well-known species and are typically recognised as their junior synonyms. However, for many of such “species” inhabiting Northern and Central Asia, names introduced in the 18^th^ and 19^th^ centuries, originally assigned to European mollusc species, were often applied. Examination of the preserved type material has revealed that these historical names were frequently misapplied, complicating the reconciliation of many published records with the modern nomenclatural system (Vinarski et al. 2013; Nekhaev et al. 2015).

Another challenge is the probable presence of true Kazakhstan (or Central Asian) endemics among the numerous species identified using the comparatory method. Most of these are reliably known from single records, and their status requires confirmation. In cases of uncertainty, such species were considered valid in our research. In general, the nomenclatural system of freshwater Mollusca adopted in this study includes the generally accepted taxa in accordance with Vinarski and Kantor (2016). Specific problematic cases are discussed in the “Results” section.

Photographs of molluscs from the ZIN collection were taken using equipment of the Core Facilities Centre “Taxon” (ZIN). Primary data processing and organisation were conducted in MS Excel. Species accumulation curves and the estimation of expected species richness using the Chao2 estimator were performed in R (version 4.4.3) with the *vegan* and *ggplot2* packages (Dixon 2003; Wickham 2011). Maps were created and visualised using QGIS (version 3.40). Photographs were processed using Pixelmator Pro 3.6.16. All data analyses and visualisations were performed on a computer equipped with an M1 Pro processor running macOS 15.3.1, using the respective software versions.

## ﻿Results

### ﻿Remarks on taxonomy and species composition of the freshwater molluscs of Kazakhstan

Despite the existence of some revisions published during recent years, not all families of freshwater Mollusca of the northern Palearctic have been revised in accordance with the integrative taxonomic approach. In total, more than 300 different names have been used by various authors to refer to molluscs from Kazakhstan. Some of these are now recognised as widely accepted synonyms, others represent misidentifications, and still others refer to species with unresolved or controversial taxonomic status. Here below, we provide some taxonomic considerations helping to understand the principles of the compiling of this check-list of Kazakhstan freshwater Mollusca. This study does not aim to conduct a taxonomic revision of any group; all comments presented above are focused primarily on the presence of particular species of molluscs within the study area.

The family Planorbidae is one of the most species-rich families of freshwater molluscs in Kazakhstan (Mozley 1936; Zhadin 1952; Vinarski et al. 2007). Apart from *Planorbis planorbis* included in our list, various authors have reported two other species of this genus, *Planorbis sieversi* Mousson, 1873, and *P. tangitarensis* Germain, 1918, from Kazakhstan (Butenko 1967; Belyakova 1980; Mazina 1988). Both species are currently considered synonyms of *Planorbis intermixtus* Mousson, 1874 (Glöer and Pešić 2010). While *P. intermixtus* has not been recorded in Kazakhstan, its range is close to the country’s borders, and its presence in the country’s southern regions is plausible (Soldatenko and Starobogatov 2000; Glöer and Pešić 2010).

We were unable to locate specimens identified as *Planorbis sieversi*, *P. tangitarensis*, or *P. intermixtus* in the ZIN collection to understand what previous authors referred to under these names. Additionally, no specimens matching the description of *Planorbis intermixtus* were found in our recent samples of the genus. Therefore, we conclude that all records of these species imply *Planorbis planorbis*.

**Figure 3.**
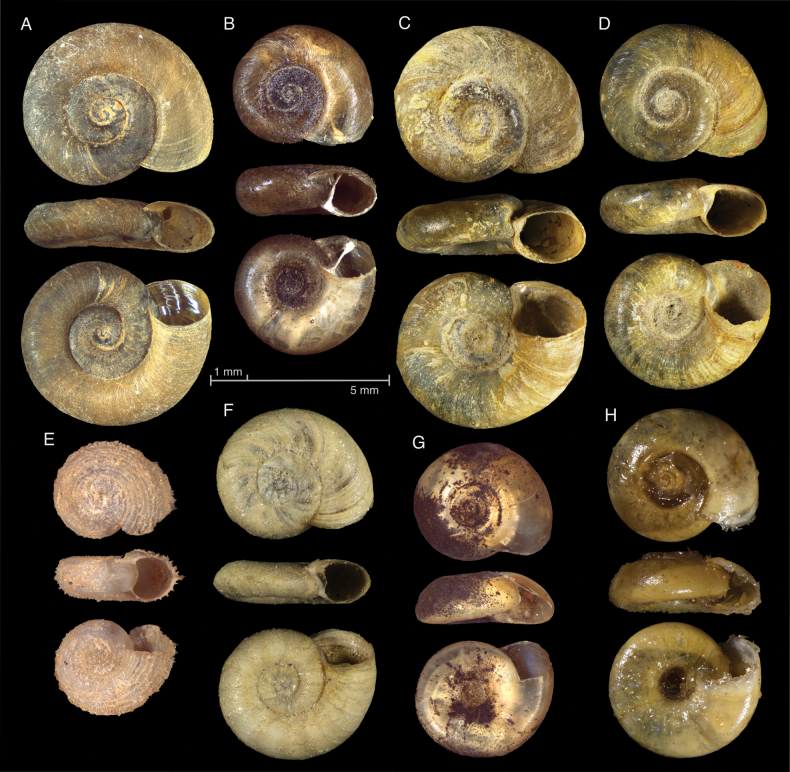
Shells of Planorbidae from Kazakhstan. A–D, F. Specimens considered here as *Gyraulus acronicus* sensu lato; E. *Gyraulus albus*, G–H. *Polypylis almaatina*; A. Irtysh Basin, near Boran Village, 47°59'57"N, 85°7'59"E, LMBI IN1186; B. Balkhash-Alakol Basin, Almaty, Shapagat, 43°17'23.35"N, 76°52’ 2.71"E, IKZ Mol-345; C, D. Irtysh Basin, Tainty Rriver, ZIN 23 (identified as *Gyraulus filiaris* by L.V. Krivosheina); E. Nura-Sarysu Basin, Nura River, 50°35'6.09"N, 70°0'11"E, LMBI 14-1328; F. Irtysh Basin, Menovnoe village, ZIN 60 (identified as *Gyraulus gredleri* by L.V. Krivosheina); G. Topotype, same locality with B, IZK Mol-344; H. holotype, Almaty, Shapagat, ZIN 1.

The inspection of samples of *Gyraulus* specimens identified by various authors as *Gyraulus filiaris* (Gredler, 1885) and *Gyraulus gredleri* (Bielz in Gredler, 1885) revealed that these names have been used at different times and by different authors to designate members of the genus lacking pronounced sculpture and keel, i.e., the traits characteristic of the species *Gyraulus acronicus* sensu lato (Nekhaev 2021) (Fig. 3A–F). Both *Gyraulus filiaris* and *Gyraulus gredleri* are excluded from the most recent list of freshwater Mollusca of the ex-USSR (Vinarski and Kantor 2016).

The only planorbid species potentially endemic to Kazakhstan, *Polypylis almaatina* Starobogatov & Mamilova, 1970, is reliably known only from water bodies within the Lake Balkhash drainage basin (Fig. 3G, H). Records of this species in the Irtysh River (Krivosheina and Starobogatov 1973) basin require further confirmation.

**Figure 4.**
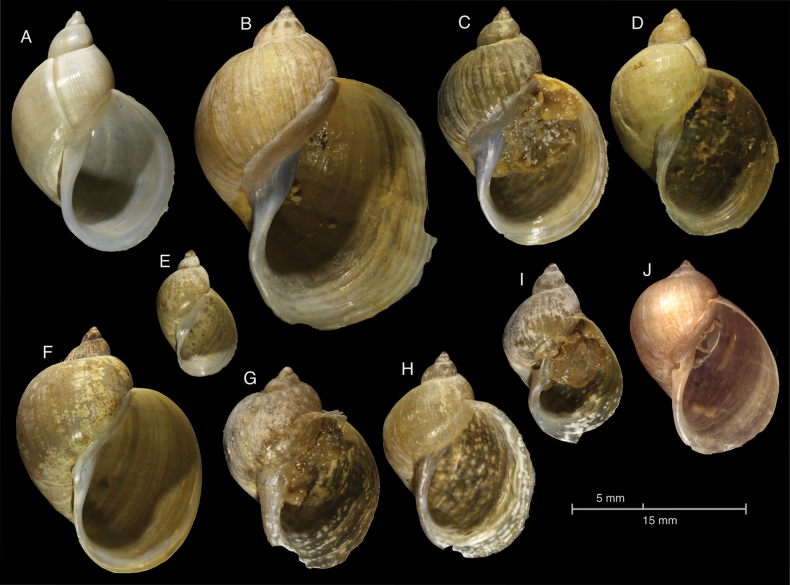
Shells of selected species of Lymnaeidae from Kazakhstan. A–I. Specimens considered here as Ampullaceana cf. lagotis from Irtysh Basin; A. Zhukey Lake, ZIN 14 (identified as *Lymnaea eversa* by A.I. Lazareva); B, C. Oskemen, ZIN 41 (identified as *Lymnaea eversa* by L.V. Krivosheina); D. Oskemen ZIN 15 (identified as *Lymnaea intermedia* by Ya.I. Starobogatov); E, F. Menovnoe Village, ZIN 74 (identified as *Lymnaea fontinalis* by L.V. Krivosheina); G–I. Kamaika River, ZIN 312 (identified as *Lymnaea peregra* by A.I. Lazareva); J. *Ampullaceana balthica*, Nura-Sarysu Basin, 50.531812°N, 69.829214°E, LMBI No. 15-2560 (identification confirmed by Cytochrome Oxidase sequence; Aksenova, pers. comm., 2020).

Among Lymnaeidae species, the greatest challenges arise in the identification and reconciliation of previous records within the subfamily Amphipepleinae Pini, 1877 (also known as ‘radicine snails’). Names such as *Lymnaea intermedia* (Lamarck, 1822), *Lymnaea lagotis* (Schrank, 1803), *Lymnaea fontinalis* (Studer, 1820), *Lymnaea peregra* (O. F. Müller, 1774), *Lymnaea eversa* (von Martens, 1882), and partially *Ampullaceana balthica* (Linnaeus, 1758) (including *Lymnaea ovata* Draparnaud, 1805) were frequently used by earlier researchers to denote conchological forms of radicine snails with relatively high spires and broad distributions across the Palearctic region. Identification of these species relied on conchometric criteria, which varied among authors (Zhadin 1952; Starobogatov et al. 2004; Kruglov 2005; Andreeva et al. 2010). Recent studies have failed to establish a direct and consistent relationship between species delineated based on shell characteristics and those identified using molecular phylogenetic data (Aksenova et al. 2018). Moreover, it has been demonstrated that records of *Ampullaceana intermedia*, *Ampullaceana fontinalis*, and *Peregriana peregra* confirmed by multigene sequence data in their interpretations by Aksenova et al. (2018) are unknown outside Europe. DNA barcoding has also revealed sporadic occurrences of *Peregriana dolgini* (Gundrizer & Starobogatov, 1979) in northern Asia, a species that is conchologically very similar to, if not indistinguishable from, the aforementioned group of species (Vinarski et al. 2015a). In most cases, it is impossible to unequivocally correlate prior identifications of molluscs in this group, made solely on the basis of shell morphology, with the contemporary taxonomic framework. Consequently, all earlier records of morphologically similar molluscs are assigned to “Ampullaceana cf. lagotis” in our list, except in cases where identification has been confirmed by molecular markers (Fig. 4).

Among the radicine pond snails with low-spired and ear-shaped shells, in addition to *Radix auricularia* (Linnaeus, 1758) (in a broad sense, encompassing *Radix bactriana* (Hutton, 1849) and *Radix intercisa* (Lindholm, 1909)), records for Kazakhstan include *Radix gebleri* (Middendorff,1850), *Lymnaea ampullacea* (Rossmaessler, 1835), and *Lymnaea tobolica* (Lazareva, 1967). The latter two species are considered junior synonyms of *Ampullaceana balthica* and *Ampullaceana ampla*, respectively. *Radix gebleri* is reliably recorded only from Lake Zaysan and is diagnosed solely based on morphology (Vinarski 2009). The shell of its holotype is significantly distinct from other members of the family, supporting its recognition as a separate species. However, reports of this species outside Lake Zaysan (e.g., Krivosheina 1979) have been attributed to *Radix auricularia* by us.

The potentially endemic species *Stagnicola iliensis* (Lazareva, 1967), of the subfamily Lymnaeinae, reported for the Ili River basin and Lake Balkhash, differs from the widely distributed *Stagnicola saridalensis* (Mozley, 1934), found in northern Central Asia and Western Siberia, only in slight proportions of their shells and the parts of the copulatory organ (Lazareva 1967; Kruglov 2005). In our study, we consider both species to be synonyms. Similarly, *Galba almaatina* (Izzatulaev, Kruglov & Starobogatov, 1983), described from the Malaya Almatinka River basin (south Kazakhstan) and differing slightly in shell proportions from the widespread *Galba truncatula*, is regarded here as a synonym of the latter. This conclusion is supported by re-examination of reproductive anatomy and analysis of COI sequences (Nekhaev et al. 2025).

Devyatkov (2011) reported a non-indigenous snail *Borysthenia naticina* (Menke, 1845) of the family Valvatidae from the water bodies of the Bukhtarma Reservoir (Upper Irtysh basin) without providing photographs or descriptions of the specimens. The materials of this species from Kazakhstan are inaccessible in the collections examined during this study. Due to its shell characteristics, this rare East European species can be easily mistaken for juvenile molluscs of the genus *Viviparus* Montfort, 1810, which have also been recorded in the same area. Therefore, we do not include *Borysthenia naticina* in the Kazakhstan fauna.

Some problems concerning the identification of valvatid snails of Kazakhstan must be noted here. A wide range of names has been used in the literature and museum collections to designate to high-spired *Valvata* species inhabiting Kazakhstan. These are *Valvata piscinalis* (O.F. Müller, 1774), *Valvata klinensis* Milaschewitsch, 1881, *Valvata depressa* Pfeiffer, 1821, *Valvata ambigua* Westerlund, 1873, *Valvata pulchella* Studer, 1789, *Valvata antiqua* Morris, 1838, and *Valvata discors* Westerlund, 1886. The distinctions between these species are typically based on shell proportions, particularly the relative height of the spire and the width of the umbilicus (Zhadin 1952; Frolova 1973; Krivosheina and Starobogatov 1973; Starobogatov et al. 2004; Vinarski et al. 2013). However, recent molecular phylogenetic studies have demonstrated that these criteria do not correspond to actual species-level differentiation (Saito et al. 2018; Schäffer and Hausdorf 2024). Therefore, we rather conservatively apply the name *Valvata piscinalis* sensu lato to designate all high-spired *Valvata* species from the region.

The species *Bithynia lindholmiana* (Starobogatov et Streletzkaja, 1967) has been reported from the Irtysh River basin (Krivosheina and Starobogatov 1973) without photographs or shell description. Hence, we attribute these records to the more widespread and conchologically similar species *Bithynia sibirica* Westerlund, 1886. Another morphologically similar species, *Bithynia caerulans* Westerlund, 1896, has been recorded from Lake Balkhash, where it is traditionally considered an endemic (Zhadin 1952; Vinarski et al. 2013). In this study, we regard *Bithynia caerulans* as a distinct species; however, more detailed investigations, including molecular phylogenetic analyses, are needed to clarify its taxonomic status.

The presence of the sphaeriid clams *Euglesa waldeni* (Kuiper, 1975) (Krivosheina 1978) and *Sphaerium nitidum* Clessin, 1876 (Krivosheina and Starobogatov 1973) in Kazakhstan has been reported; however, the authors did not provide any photographs or drawings. Both species are reliably known only from the northern Holarctic (Kuiper 1975; Clarke 1981; Kuiper et al. 1989; Vinarski and Kantor 2016; Vinarski et al. 2021a; Bespalaya et al. 2024), the former also from the mountainous regions of eastern Siberia (Bespalaya et al. 2024) and from Japan (Lee and Ó Foighil 2003; Saito et al. 2022). The close similarity of the supposed *Euglesa waldeni* with *Euglesa lilljeborgii* (Clessin, 1886), which has been repeatedly found in Kazakhstan, is directly indicated by Krivosheina (1978); therefore, we consider this find to be *Euglesa lilljeborgii*. *Sphaerium nitidum* is listed with the widespread and repeatedly encountered in Kazakhstan species *Sphaerium corneum* and *Sphaerium nucleus*. Therefore, we do not include species *Euglesa waldeni* and *Sphaerium nitidum* in our species list.

The taxonomic status of fingernail clams recorded from Kazakhstan by Frolova (1973) under the name “*Euglesa unionioides*” (most probably, it referred to Pisidium milium var. unioides Westerlund, 1873) also remains uncertain. That taxon was originally described from northern Scandinavia (Westerlund, 1890), and we were unable to locate any collection materials that would allow us to verify the validity of this record.

Belyakova (1980) reported the presence of *Anadonta cellensis* Pfeiffer, 1821 in the water bodies of the Balkhash–Alakol Basin. In current nomenclature, this name is considered a synonym of *Anodonta cygnea*, a species reliably known only from Europe (Bolotov et al. 2020). Therefore, we consider it most likely that Belyakova’s records refer to *Anodonta anatina*, a species widely distributed across Kazakhstan.

### ﻿Estimated species composition

**Figure 5.**
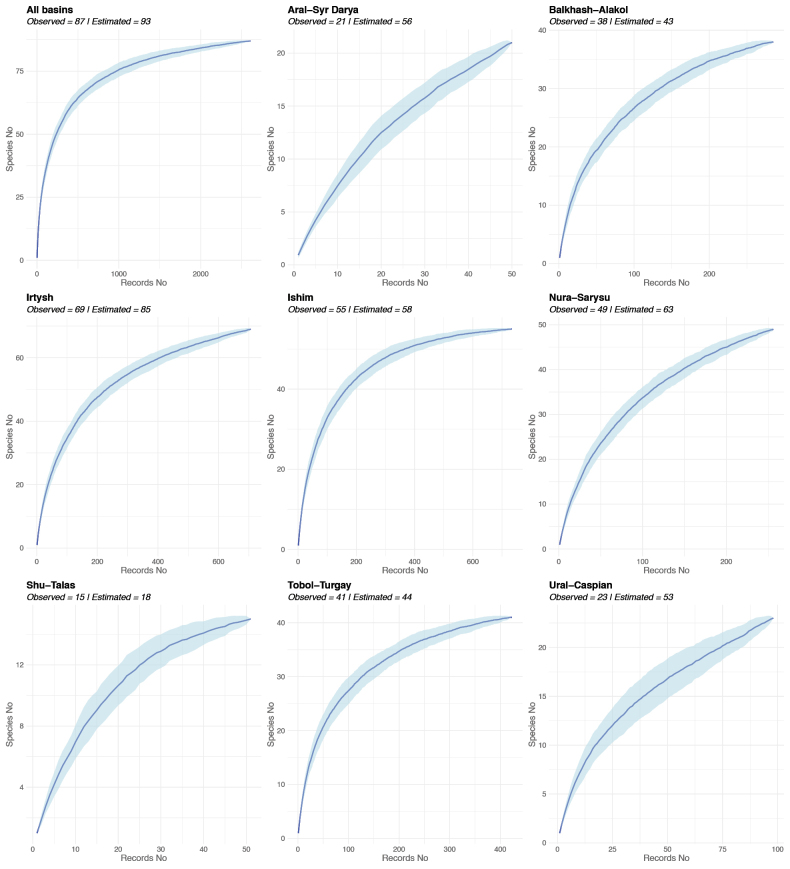
Species accumulation curves and estimated species richness based on the Chao2 estimator for the entire territory of Kazakhstan and its major drainage basins. The shaded area indicates the 95% confidence interval. The X-axis represents the number of records in our database; the Y-axis indicates the number of recorded species.

The compiled list (Table 1) includes 87 species of freshwater molluscs, comprising 55 gastropod and 32 bivalve species. The distribution of species richness across drainage basins and the completeness of its assessment are presented in Fig. 5. The Irtysh and Ishim drainage basins exhibit the highest species richness, whereas the Shu–Talas, Ural–Caspian, and Aral–Syr Darya basins maintain the least diversity. Overall, the freshwater malacofauna of Kazakhstan has been documented with satisfactory completeness. The most comprehensive data have been obtained for the Ishim and Tobol–Turgay basins, where the estimated number of species closely approaches the observed richness. However, the mollusc fauna of most other basins, particularly those in the western and southern regions of the country, is likely understudied.

**Table 1.** Species composition and number of finds of freshwater molluscs in the main drainage basins of Kazakhstan.

Species	Aral-Syr Darya	Balkhash-Alakol	Shu-Talas	Ural-Caspian	Irtysh	Ishim	Nura-Sarysu	Tobol-Turgay
Bivalvia								
Family Cardiidae								
*Monodacna colorata* (Eichwald, 1829)	–	4	–	–	–	–	–	–
Family Cyrenidae								
*Corbicula fluminalis* (O. F. Müller, 1774)	1	1	–	–	–	–	–	–
*Corbicula tibetensis* Prashad, 1929	1	–	2	–	–	–	–	–
Family Dreissenidae								
*Dreissena polymorpha* (Pallas, 1771)	3	1	–	1	–	–	–	–
Family Sphaeridae								
*Conventus conventus* (Clessin, 1877)	–	–	–	–	1	2	1	–
*Euglesa casertana* (Poli, 1791)	–	2	–	–	26	6	2	2
*Euglesa globularis* (Clessin, 1873)	1	–	–	–	8	–	–	–
*Euglesa henslowana* (Sheppard, 1825)	–	2	–	–	12	6	2	2
*Euglesa lilljeborgii* (Clessin, 1886)	–	–	–	–	4	3	1	–
*Euglesa milium* (Held, 1836)	–	–	–	–	3	3	1	–
*Euglesa nitida* (Jenyns, 1832)	–	1	1	–	6	–	–	–
*Euglesa obtusalis* (Lamarck, 1818)	–	–	–	–	6	3	1	–
*Euglesa parvula* (Westerlund, 1873)	–	–	–	–	2	–	–	–
*Euglesa ponderosa* (Stelfox, 1918)	–	–	–	–	2	–	–	–
*Euglesa pulchella* (Jenyns, 1832)	–	–	–	–	4	1	–	–
*Euglesa subtruncata* (Malm, 1855)	1	2	–	–	24	6	2	1
*Euglesa supina* (Schmidt, 1851)	–	–	–	–	1	–	–	–
*Odhneripisidium dancei* (Kuiper, 1962)	–	–	–	–	1	–	–	–
*Odhneripisidium moitessierianum* (Paladilhe, 1866)	–	–	–	–	5	–	–	–
*Odhneripisidium stewarti* (Preston, 1909)	–	2	–	–	1	–	–	–
*Odhneripisidium tenuilineatum* (Stelfox, 1918)	–	–	–	–	5	–	–	–
*Pisidium amnicum* (O. F. Müller, 1774)	–	1	–	–	4	3	1	1
*Sphaerium asiaticum* (von Martens, 1864)	–	–	–	–	2	1	–	–
*Sphaerium corneum* (Linnaeus, 1758)	–	2	–	–	8	7	2	2
*Sphaerium lacustre* (O. F. Müller, 1774)	–	3	–	–	9	4	1	1
*Sphaerium nucleus* (Studer, 1820)	–	–	–	–	2	–	–	–
*Sphaerium rivicola* (Lamarck, 1818)	–	–	–	–	–	–	–	1
Family Unionidae								
*Anodonta anatina* (Linnaeus, 1758)	4	3	1	1	44	11	5	6
*Anodonta cygnea* (Linnaeus, 1758)	–	3	–	–	–	–	–	–
*Sinanodonta lauta* (von Martens, 1877)	–	3	–	–	–	–	–	–
*Sinanodonta woodiana* (Lea, 1834)	1	3	–	–	–	–	–	–
*Unio crassus* Philipsson, 1788	–	–	–	1	–	–	–	–
*Unio tumidus* Philipsson, 1788	–	–	–	–	6	–	–	–
Gastropoda								
Family Bithyniidae								
*Bithynia boissieri* (Küster, 1852)	–	–	–	–	–	4	1	3
*Bithynia caerulans* Westerlund, 1896	–	10	1	–	2	–	–	–
*Bithynia sibirica* Westerlund, 1886	–	–	–	1	3	–	2	–
*Bithynia tentaculata* (Linnaeus, 1758)	–	–	–	–	1	36	4	22
*Bithynia transsilvanica* (Bielz, 1853)	–	–	–	1	13	29	13	32
Family Hydrobiidae								
*Martensamnicola brevicula* (von Martens, 1874)	1	–	–	–	–	–	–	–
*Martensamnicola kazakhstanica* Izzatullaev, Sitnikova & Starobogatov, 1985	2	–	–	–	–	–	–	–
*Sibirobythinella almaatina* Izzatullaev, Sitnikova & Starobogatov, 1985	–	1	–	–	–	–	–	–
Family Viviparidae								
*Viviparus viviparus* (Linnaeus, 1758)	–	–	–	–	2	–	–	–
Family Lithoglyphidae								
*Lithoglyphus naticoides* (Pfeiffer, 1828)	–	–	–	–	2	–	–	–
Family Valvatidae								
*Valvata cristata* O. F. Müller, 1774	–	–	–	–	1	5	1	1
*Valvata macrostoma* Mörch, 1864	–	–	–	–	1	–	–	–
*Valvata piscinalis* (O. F. Müller, 1774)	1	16	–	2	22	106	3	48
*Valvata sibirica* Middendorff, 1851	–	–	–	–	12	6	2	2
Family Acroloxidae								
*Acroloxus lacustris* (Linnaeus, 1758)	–	1	–	1	2	3	1	2
Family Lymnaeidae								
*Aenigmomphiscola uvalievae* Kruglov & Starobogatov, 1981	–	–	–	–	–	14	4	–
*Ampullaceana ampla* (Hartmann, 1821)	–	–	–	2	1	3	3	6
*Ampullaceana balthica* (Linnaeus, 1758)	–	–	–	–	28	52	23	22
Ampullaceana cf. lagotis (Schrank, 1803)	7	22	11	5	88	66	18	26
*Galba truncatula* (O. F. Müller, 1774)	3	18	4	3	11	4	4	5
*Kazakhlymnaea taurica kazakensis* (Mozley, 1934)	1	2	4	5	8	11	13	32
*Ladislavella terebra* (Westerlund, 1885)	–	4	4	–	14	9	5	11
*Lymnaea stagnalis* (Linnaeus, 1758)	4	29	6	13	53	66	32	52
*Myxas glutinosa* (O. F. Müller, 1774)	–	–	–	–	1	2	1	7
*Orientogalba heptapotamica* (Lazareva, 1967)	–	6	2	–	1	–	1	–
*Peregriana dolgini* (Gundrizer & Starobogatov, 1979)	–	–	–	–	1	–	–	–
*Radix auricularia* (Linnaeus, 1758)	6	42	5	9	79	36	17	28
*Radix gebleri* (Middendorff, 1850)	–	–	–	–	3	–	–	–
*Stagnicola palustris* (O. F. Müller, 1774)	–	1	–	9	4	14	4	7
*Stagnicola saridalensis* (Mozley, 1934)	1	17	4	5	8	17	14	15
Family Physidae								
*Aplexa hypnorum* (Linnaeus, 1758)	–	2	–	1	3	4	3	–
*Physa fontinalis* (Linnaeus, 1758)	–	–	–	1	–	11	4	4
*Physa taslei* Bourguignat, 1860	–	–	–	–	5	1	2	2
*Physella acuta* (Draparnaud, 1805)	1	14	–	–	6	3	2	–
*Sibirenauta elongata* (Say, 1821)	–	–	–	–	1	3	–	–
Family Planorbidae								
*Ancylus fluviatilis* O. F. Müller, 1774	–	–	–	–	1	–	–	–
*Anisus leucostoma* (Millet, 1813)	–	–	–	–	–	2	–	2
*Anisus spirorbis* (Linnaeus, 1758)	–	–	–	–	4	11	3	4
*Anisus vortex* (Linnaeus, 1758)	1	2	–	–	4	10	3	6
*Anisus vorticulus* (Troschel, 1834)	–	–	–	–	4	7	1	3
*Armiger bielzi* (Kimakowicz, 1884)	–	–	–	–	1	4	3	4
*Armiger crista* (Linnaeus, 1758)	–	–	–	–	5	3	1	–
*Bathyomphalus contortus* (Linnaeus, 1758)	–	3	–	–	10	19	3	3
*Gyraulus acronicus* (Férussac, 1807)	4	15	1	16	47	29	4	5
*Gyraulus albus* (O. F. Müller, 1774)	–	7	1	1	3	9	3	5
*Gyraulus parvus* (Say, 1817)	–	–	–	–	8	4	1	–
*Gyraulus riparius* (Westerlund, 1865)	–	–	–	–	–	1	–	1
*Gyraulus rossmaessleri* (Auerswald, 1852)	–	–	–	1	1	2	–	–
*Gyraulus stroemi* (Westerlund, 1881)	–	–	–	–	–	5	1	–
*Hippeutis complanatus* (Linnaeus, 1758)	1	–	–	–	3	3	–	3
*Planorbarius corneus* (Linnaeus, 1758)	–	1	–	1	15	17	8	9
*Planorbis planorbis* (Linnaeus, 1758)	1	11	3	13	10	21	22	30
*Polypylis almaatina* Starobogatov & Mamilova, 1970	–	4	–	–	1	–	–	–
*Segmentina nitida* (O. F. Müller, 1774)	–	1	–	5	7	17	4	5

## ﻿Discussion

The data obtained on the species composition of freshwater molluscs in Kazakhstan represent the most comprehensive and verifiable inventory of a freshwater malacofauna of Central Asian countries to date. However, our study has several limitations that may lead to an underestimation of the actual taxonomic diversity of molluscs in the country. First, assessing the true sampling coverage across different regions of Kazakhstan remains challenging. The relatively low number of mollusc records from desert and mountainous areas is most likely due to their naturally low occurrence in these habitats. However, species richness in these regions may also be underestimated due to their inaccessibility to researchers.

A second potential factor of underestimation arises from the conservative approach to mollusc classification applied in this study. The use of operational taxonomic units or “morphospecies” – which do not always fully correspond to actual biological species – may be the only viable method for inventorying the fauna of large invertebrate groups. Nevertheless, studies employing molecular phylogenetic methods have demonstrated that cryptic and pseudocryptic species are relatively common among freshwater molluscs, including the groups represented in the Kazakhstan fauna (Pfenninger et al. 2003; Kulsantiwong et al. 2013; Aksenova et al. 2018, 2024; Vinarski 2024).

Finally, our study does not provide a detailed assessment of species that may inhabit both freshwater and brackish environments (e.g., some Cardiidae, Dreissenidae, Hydrobiidae), as we included only those whose presence in freshwater ecosystems is firmly known. The brackish-water fauna of Central Asia requires a separate analysis and falls outside the scope of this study.

The overall diversity of freshwater molluscs in Kazakhstan is relatively high and significantly exceeds the known diversity in Mongolia and Kyrgyzstan, where only 35 and 27 species of gastropods, respectively, have been recorded (Glöer et al. 2014; Vinarski et al. 2017a) (the comparable data on freshwater clams of these countries are unavailable). This may be at least partly explained by the fact that the territory of Kazakhstan is much larger than the area of ​​these two states. However, the diversity of freshwater Mollusca observed in Kazakhstan is also only slightly lower than that of adjacent, more extensive regions with greater habitat heterogeneity, such as Western Siberia (90 gastropod species, 29 bivalve species) and Central Siberia (91 gastropod species, 31 bivalve species) (Vinarski and Kantor 2016).

In general, the geographic patterns of freshwater mollusc species richness align with landscape-zonal regionalisation of the Kazakhstan territory. The highest species richness is observed in the forest and forest-steppe zones, it decreases in the steppe zone and reaches its lowest levels in desert and mountainous regions (southern and southwestern Kazakhstan). A decline in freshwater mollusc diversity along the gradient from forested to treeless tundra landscapes was previously documented by us in northern Europe (Nekhaev 2021). This pattern may be linked to a reduction in the influx of terrestrial organic matter as leaf litter, which often serves as a food source for molluscs.

In Kazakhstan, however, another significant factor influencing mollusc diversity is the sharp decrease in precipitation from north to south, leading to a corresponding reduction in the diversity of freshwater habitats (Salnikov et al. 2023). This decline in available aquatic environments may play a crucial role in the reduction of taxonomic diversity. A similar explanation was earlier proposed by Vinarski et al. (2012a) in their attempt to understand the decline of freshwater mollusc diversity in the south of Western Siberia.

Overall, the freshwater malacofauna of Kazakhstan consists primarily of species widely distributed across the Palearctic, which also represent the most frequently encountered taxa across different ecoregions and drainage basins of the studied country. Notably, there are no endemic species in Kazakhstan whose taxonomic status has been confirmed by molecular phylogenetic data. A partial exception may be the subspecies *Kazakhlymnaea taurica kazakensis*, whose distribution is largely confined to Kazakhstan but also extends into the southern regions of western Siberia (Vinarski et al. 2012b).

The taxonomic status of six species (*Bithynia caerulans*, *Martensamnicola brevicula*, *Martensamnicola kazakhstanica*, *Sibirobythinella almaatina*, *Polypylis almaatina*, and *Radix gebleri*), for which no confirmed records exist outside Kazakhstan, requires further verification. None of them has ever been subjected to a molecular analysis which would confirm (or reject) their species status. Unfortunately, one of these putative endemics, *Radix gebleri*, may have already become extinct as a result of the creation of the Bukhtarma Reservoir in the 1960s, which drastically altered the hydrological regime of Lake Zaysan. A special search conducted in July 2018 by one of the authors did not result in the finding of either living individuals or even empty shells of *Radix gebleri* in this waterbody. On the other hand, we hypothesise that further research may reveal a greater number of endemic species, particularly among the microgastropods of the family Hydrobiidae, as has been observed in other mountainous regions (Chertoprud et al. 2020, 2023).

At least seven species of freshwater molluscs in the fauna of Kazakhstan may be classified as neobiotic. Among them, the East Asian bivalve species *Corbicula fluminalis*, *Sinanodonta woodiana*, *Sinanodonta lauta* and the North American snail *Physella acuta* have widely expanded their ranges across various regions of the Palearctic or even globally due to human activities (Sousa et al. 2008; Kondakov et al. 2020; Nekhaev et al. 2024). We propose a similar colonisation mechanism for these species in Kazakhstan as well.

Relatively recently, populations of the European species *Unio tumidus* were recorded in Kazakhstan and adjacent parts of Western Siberia (Babushkin et al. 2021). Molecular phylogenetic analyses have revealed unique haplotypes in these populations, which likely indicate the ongoing recolonisation of the once lost regions from a Quaternary refugium. A similar hypothesis has been proposed for two gastropod species, *Lithoglyphus naticoides* and *Viviparus viviparus*, which were found in sympatry with *Unio tumidus* in the upper reaches of the Irtysh River (Babushkin et al. 2023). These species are known from Quaternary deposits in southern Western Siberia and are likely restoring the part of their former ranges which were lost as a result of the Pleistocene glaciation events (Babushkin et al. 2023).

In addition to the neobiotic species included in the list as forming stable populations, a single empty shell of the lymnaeid *Pseudosuccinea columella* (Say, 1817) was found within the territory of Kazakhstan (Vinarski et al. 2015b). However, this species has not been subsequently recorded from this or any other region of Kazakhstan. *Pseudosuccinea columella* is a widespread species in the aquarium trade of American origin, and the introduction of empty shells or live individuals of this and other aquarium species into natural water bodies is sporadically possible. Nevertheless, such introductions often do not result in the establishment of viable populations.

## ﻿Conclusions

This study presents the first comprehensive synthesis of freshwater mollusc diversity in Kazakhstan, revealing a total of 87 confirmed species and an estimated maximum of 93. The freshwater malacofauna is dominated by widespread Palearctic taxa, but also includes several putative endemics whose status requires molecular confirmation. Species richness displays a clear latitudinal gradient, declining from the forest-steppe zones in the north to the arid and mountainous regions in the south and southwest, a pattern likely driven by both climatic aridification and reduced freshwater habitat availability.

While the current inventory significantly advances our knowledge of Kazakhstan’s freshwater molluscs, it also highlights the limitations of species-level resolution in poorly studied regions. The presence of at least seven neobiotic species, some of which may be recolonising habitats lost during the Pleistocene, underscores the role of both anthropogenic dispersal and long-term biogeographic processes in shaping contemporary faunal assemblages.

Future research should prioritise field surveys in under-sampled areas, integration of genetic data for species delimitation, and a separate assessment of the brackish-water molluscan fauna. These efforts are essential for capturing the full extent of Kazakhstan’s freshwater mollusc diversity and informing conservation priorities across its diverse aquatic ecosystems.

## ﻿Additional information

### Use of AI

No use of AI was reported.

### Author ORCIDs

Ivan O. Nekhaev https://orcid.org/0000-0002-9464-715X

Anel A. Ishayeva https://orcid.org/0009-0009-9578-4736

Maxim V. Vinarski https://orcid.org/0000-0002-7644-4164

Evgeniy S. Babushkin https://orcid.org/0000-0002-9980-189X

### Data availability

All of the data that support the findings of this study are available in the main text or Supplementary Information.

## Supplementary materials

Supplementary material 1 The original database on freshwater mollusks of Kazakhstan Data type xlsx Explanation note It includes all recorded occurrences, their sources, and geographic coordinates, as well as the original species names used in the sources and the recommended standardized names. The second sheet provides a table of the referenced literature. https://binary.pensoft.net/file/1402488This dataset is made available under the Open Database License (http://opendatacommons.org/licenses/odbl/1.0/). The Open Database License (ODbL) is a license agreement intended to allow users to freely share, modify, and use this Dataset while maintaining this same freedom for others, provided that the original source and author(s) are credited.Ivan O. Nekhaev, Anel A. Ishayeva, Maxim V. Vinarski, Evgeniy S. Babushkin
